# Fruquintinib-Induced Cerebellar Hemorrhage in Left-Sided Descending Metastatic Colorectal Adenocarcinoma: A Case Report and Risk Assessment

**DOI:** 10.7759/cureus.68203

**Published:** 2024-08-30

**Authors:** Daniel T Jones, Gabriel Cruz, Maria Theresa Lugue, Manvir S Heer, Marlai Sai, Christopher Pace, Linsey Bui, Scott A Silver

**Affiliations:** 1 Internal Medicine, Touro University Nevada, Henderson, USA; 2 Internal Medicine, Touro University California, Vallejo, USA; 3 Internal Medicine, Kansas City University, Joplin, USA; 4 Neurology, Valley Hospital Medical Center Neurology Residency Program, Las Vegas, USA; 5 Internal Medicine, Valley Hospital Medical Center Internal Medicine Residency Program, Las Vegas, USA

**Keywords:** chemotherapy, targeted therapy, vegfr inhibitor, cerebellar hemorrhage, side effects, fruzaqla, fruquintinib, metastatic colorectal cancer (mcrc), metastatic colorectal adenocarcinoma

## Abstract

Colorectal adenocarcinoma is the most prevalent form of colorectal cancer, representing the majority of cases in the United States. The disease is driven by a series of genetic mutations, including alterations in the adenomatous polyposis coli (APC), Kirsten rat sarcoma viral oncogene homolog G12D (KRAS), human epidermal growth factor receptor 2 immunohistochemistry 3+ (HER-2 IHC3+), checkpoint kinase 2 (CHEK-2) and tumor protein P53 (TP53) genes, which lead to malignant transformation. While the standard treatment for metastatic colorectal cancer (mCRC) typically involves chemotherapy and targeted therapies, many patients experience disease progression, necessitating the exploration of novel treatments. Fruquintinib, a highly selective vascular endothelial growth factor (VEGFR) inhibitor, has emerged as a promising option for mCRC patients who have exhausted conventional therapies. However, its use is associated with significant bleeding risks, including rare but severe complications such as cerebellar hemorrhage. This case report presents a patient with mCRC who developed a cerebellar hemorrhage shortly after initiating fruquintinib therapy, highlighting the need for careful patient monitoring and individualized risk assessment to mitigate such serious adverse events.

## Introduction

Colorectal adenocarcinoma is the most prevalent form of colorectal cancer, originating from the glandular cells lining the colon and rectum [1]. The development of this cancer involves a sequence of genetic mutations, particularly in the adenomatous polyposis coli (APC), Kirsten rat sarcoma viral oncogene homolog G12D (KRAS), human epidermal growth factor receptor 2 immunohistochemistry 3+ (HER-2 IHC3+), checkpoint kinase 2 (CHEK-2) and tumor protein P53 (TP53) genes, which lead to the malignant transformation of normal epithelial cells [2]. Several risk factors contribute to colorectal adenocarcinoma, including a family history of the disease, hereditary syndromes such as Lynch syndrome and familial adenomatous polyposis (FAP), as well as lifestyle factors such as a diet high in red and processed meats, smoking, alcohol use, obesity, and physical inactivity [3]. Although colorectal adenocarcinoma predominantly affects individuals over 50, there is a concerning increase in incidence among younger adults, likely driven by a combination of genetic predisposition, dietary changes, increased antibiotic use, and obesity [4]. Common clinical presentations include changes in bowel habits, rectal bleeding, abdominal pain, and unexplained weight loss. Diagnosis is typically confirmed through colonoscopy and histological examination, followed by staging to guide treatment [5].

The treatment of metastatic colorectal cancer (mCRC) is challenging, often involving chemotherapeutic agents such as fluoropyrimidines, oxaliplatin, and irinotecan, combined with targeted therapies like anti-vascular endothelial growth factor (VEGF) (bevacizumab) and anti-epidermal growth factor receptor agents (cetuximab, panitumumab) [6]. However, disease progression despite these therapies is common, necessitating new treatment options. Fruquintinib, which specifically targets VEGFR1, VEGFR2, and VEGFR3, is more selective in its action against angiogenesis and generally has fewer side effects compared to bevacizumab, which targets the broader VEGF-A, affecting multiple pathways and potentially leading to a wider range of side effects [7]. Initially approved in China in 2018 and more recently in Europe and the United States, fruquintinib has shown efficacy in prolonging progression-free survival in patients who have exhausted conventional treatments in mCRC [8]. Despite its benefits, fruquintinib carries risks associated with VEGFR inhibition, including hypertension, gastrointestinal side effects, and, more rarely, severe hemorrhagic events [9].

This case report discusses a patient with mCRC who developed a cerebellar hemorrhage following the initiation of fruquintinib therapy. Hemorrhagic complications, while recognized with VEGFR inhibitors, are rare and particularly concerning when occurring in the cerebellum. This case emphasizes the importance of vigilant monitoring and individualized risk assessment when using newer targeted therapies. The objective of this report is to highlight the potential risks associated with fruquintinib and contribute to the growing body of knowledge on its safety profile.

## Case presentation

**Figure 1:**
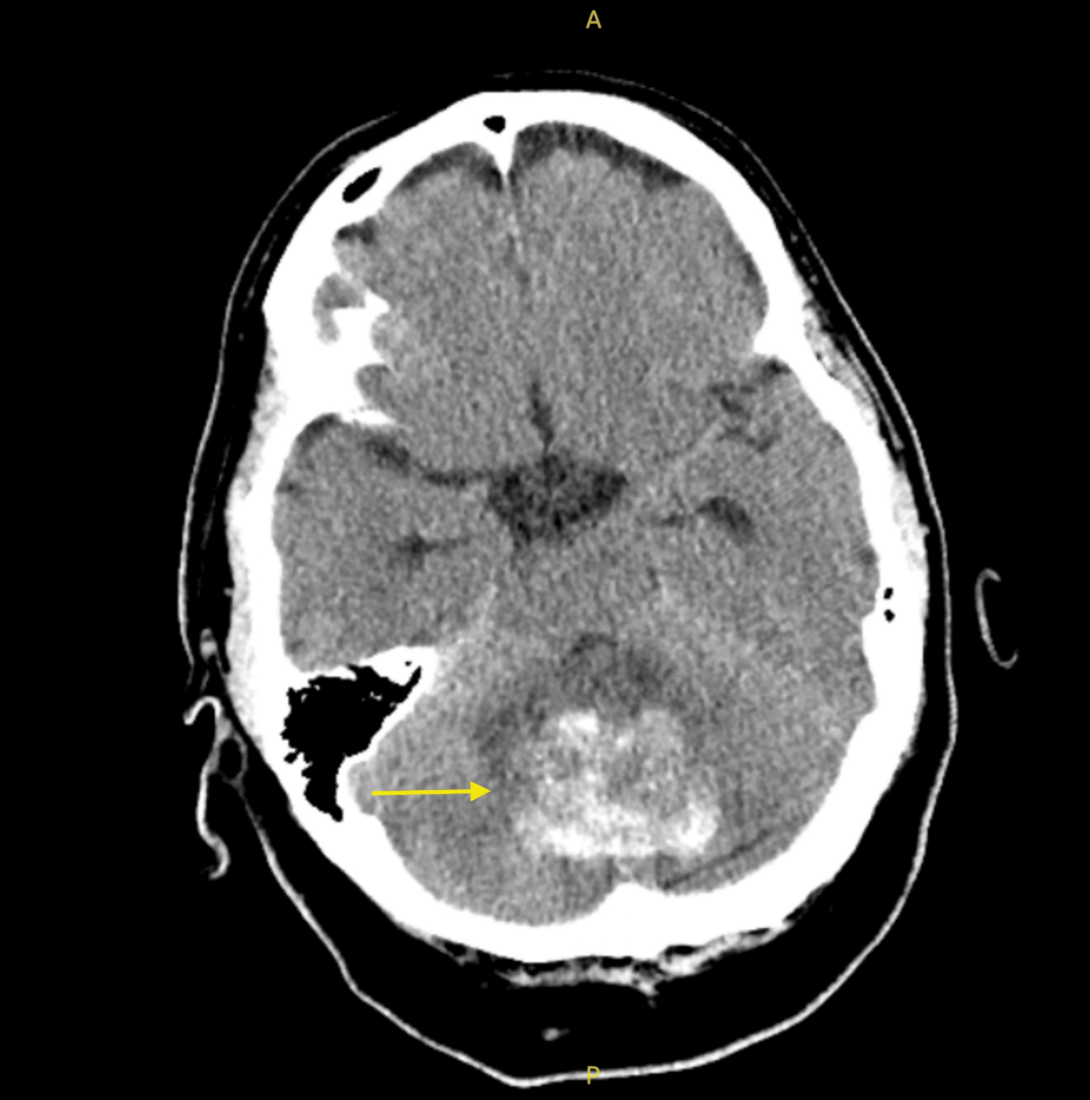
Axial non-contrast CT brain showing a 4.4 cm hemorrhagic cerebellar mass (yellow arrow) with mass effect on the fourth ventricle and early hydrocephalus The letters "A" and "P" indicate the anterior and posterior aspects of the image, respectively. Image Credit: Daniel Jones

The patient, a 46-year-old Caucasian male, has a medical history of stage IV left-sided descending colorectal adenocarcinoma with metastasis to the lungs, diagnosed in 2018. The patient is currently visiting town on vacation. His cancer is associated with a CHEK2 gene mutation and is HER-2 IHC3+ and KRAS G12D positive. He previously underwent colon resection with colostomy and received the FOLFOX regimen, which consists of folinic acid (leucovorin), fluorouracil (5-FU), and oxaliplatin. The patient did not respond to the FOLFOX regimen and was then put on trifluridine and tipiracil (Lonsurf), starting at 35 mg/m² up to a maximum of 80 mg per dose. The patient did not respond to trifluridine and tipiracil. He was then put on fruquintinib, which included 5 mg of fruquintinib daily. The patient's only other medication was Percocet 5-325 every six hours as needed for the cancer pains. The fruquintinib regimen consists of a 28-day cycle, during which the patient takes the medication for the first 21 days followed by a seven-day break. On day 18 of his first cycle of fruquintinib, he began experiencing new symptoms, including an unsteady gait, confusion with word-finding difficulties, and a persistent left-sided headache. He denied any recent falls, memory impairment, head trauma, or other neurological symptoms like light sensitivity or extremity numbness. Believing fruquintinib to be the cause, the patient discontinued the drug after 18 doses in his first cycle. Upon arrival at the emergency room, the patient’s vital signs included a blood pressure of 122/86 mmHg and a heart rate of 104 bpm. A CT scan of the head revealed a 3.2 x 4.2 x 2.9 cm cerebellar hemorrhage with a significant localized mass effect, resulting in the effacement of the basilar cisterns. Additionally, ventriculomegaly was noted, with periventricular hypodensity suggesting potential transependymal flow of cerebrospinal fluid (CSF), raising concerns about increased intracranial pressure and the possibility of early hydrocephalus. No additional intra-axial or extra-axial bleeding was observed, and the gray-white matter interface remained preserved (Figure 1).

**Figure 2:**
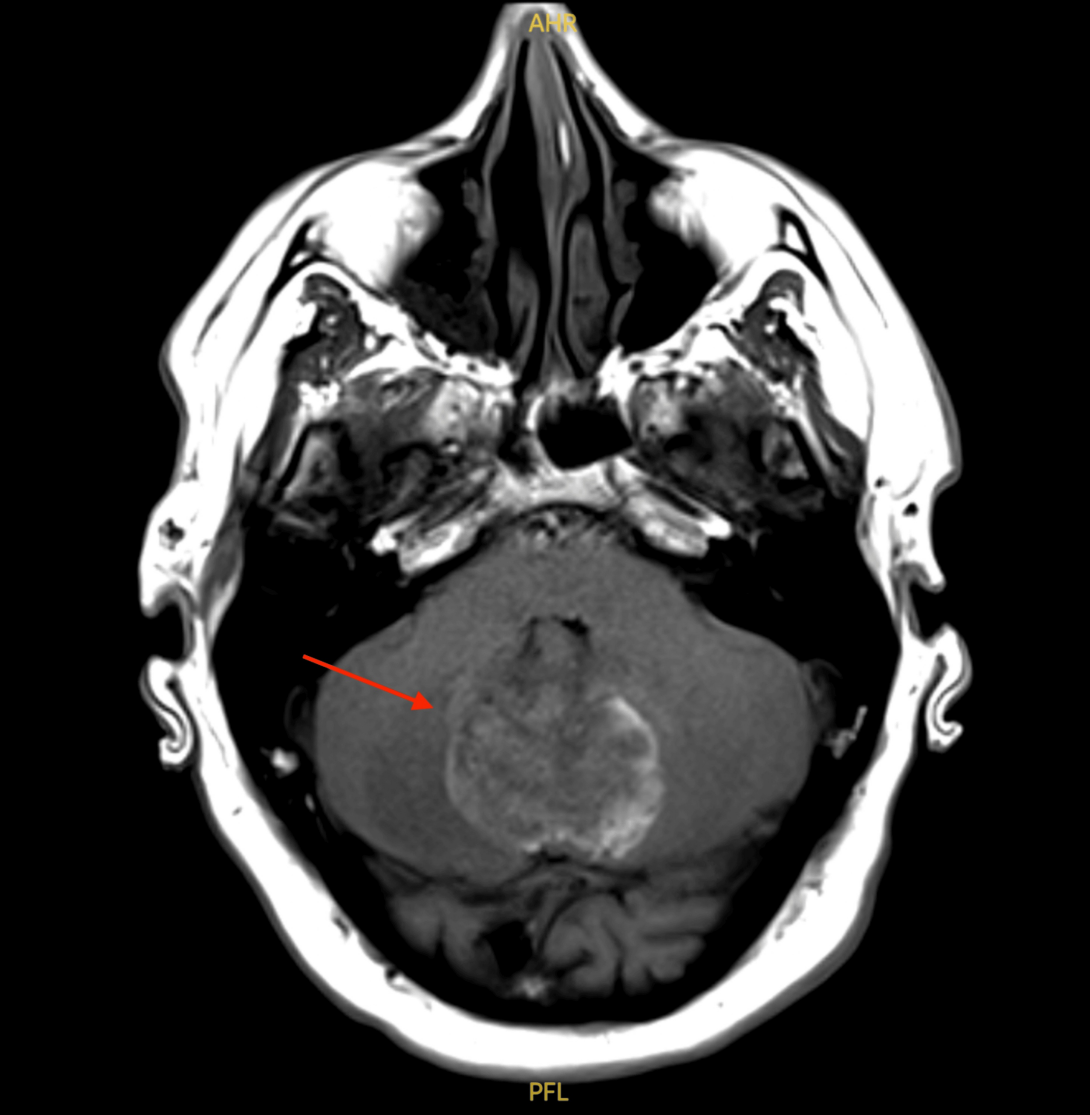
Axial post-contrast MRI showing a 4.4 cm hemorrhagic midline posterior fossa mass (red arrow) with mild enhancement and mass effect on the fourth ventricle Anterior horn of lateral ventricle (AHR) and posterior fossa lesion (PFL) are annotated in the image. Image Credit: Daniel Jones

An MRI of the brain confirmed a midline posterior fossa mass in the cerebellum, showing hemorrhagic characteristics with mild mass effect upon the fourth ventricle. Surrounding edema was also noted, with minimal dilation of the lateral and third ventricles, consistent with early hydrocephalus. Post-contrast imaging revealed mild enhancement of the mass, with no other enhancing masses detected in the brain (Figure 2).

The non-contrast CT and MRI findings led to the suspicion that the cerebellar hemorrhage might be related to fruquintinib therapy, prompting urgent consultations with neurology and neurosurgery. Neurology recommended no antiplatelets or anticoagulants at this time, maintaining systolic blood pressure (SBP) goals below 150, and considering palliative care. Per neurosurgery, no surgical intervention for the bleed or external ventricular drain (EVD) is to be placed. Neurosurgery did recommend continued monitoring for potential hydrocephalus. The patient was admitted to the intensive care unit (ICU) for close monitoring, including hourly neurological checks, given the severity of the hemorrhage and the associated risk of further complications.

The patient's family expressed a desire to speak to hospice as they wished to return to their home state. On ICU day 4, before the hospice consultation, the patient decided to leave against medical advice (AMA) with his family. The patient was alert and oriented x4, ambulatory, and in no apparent distress at the time of departure. Despite an explanation of his current medical condition and the risks associated with leaving, including but not limited to worsening symptoms, chronic condition progression, and the possibility of death, the patient expressed understanding but ultimately chose to leave AMA. Pending orders and imaging were not able to be completed. He was instructed to follow up with his primary care provider and to return to the emergency department for any new or worsening symptoms.

## Discussion

Colorectal adenocarcinoma is the most common type of colorectal cancer, originating from the glandular cells lining the colon and rectum [1]. In the United States, over 150,000 new cases of colorectal cancer are diagnosed each year, with adenocarcinoma accounting for the majority of these cases [1]. Approximately 20% of these patients present with metastatic disease at the time of diagnosis, classifying them as stage IV [2]. This advanced stage is characterized by the spread of cancer to distant organs, most commonly the liver and lungs, and is associated with a significantly lower five-year survival rate compared to earlier stages [3]. The development of colorectal adenocarcinoma typically involves a series of genetic mutations, including those in the APC, KRAS, and TP53 genes, as well as epigenetic changes that transform normal colonic epithelium into adenomatous polyps and eventually invasive carcinoma [4].

The patient’s genetic profile, including a CHEK2 mutation, HER-2 positivity, and a KRAS mutation, significantly impacts the behavior of colorectal adenocarcinoma and its response to treatment. KRAS mutations, present in approximately 40% of colorectal cancers, are associated with resistance to EGFR inhibitors, complicating treatment [5]. The CHEK2 mutation, which impairs DNA repair mechanisms, further increases the patient’s risk for cancer development [6]. HER-2 positivity, although more commonly associated with breast cancer, can drive tumor aggressiveness in colorectal cancer, making treatment more challenging. HER-2-positive, targeted therapies such as trastuzumab, pertuzumab, and lapatinib, often used in combination with chemotherapy, offer an effective alternative for patients resistant to standard treatments [7].

Fruquintinib is an orally administered, highly selective inhibitor of vascular endothelial growth factor receptors (VEGFR) 1, 2, and 3. It is approved for the treatment of mCRC in patients who have exhausted standard treatment options, including fluoropyrimidines, oxaliplatin, irinotecan, and targeted therapies such as anti-VEGF and anti-EGFR agents [8]. Initially approved in China in 2018, fruquintinib has since gained global recognition, particularly following clinical trials like FRESCO and FRESCO-2, which demonstrated significant improvements in PFS for heavily pretreated mCRC patients compared to placebo [9].

The FRESCO trial, a pivotal phase III study, demonstrated that fruquintinib significantly improved PFS and overall survival (OS) compared to placebo in patients with mCRC who had been previously treated with standard therapies. Specifically, the median PFS was 3.7 months for fruquintinib compared to 1.8 months for placebo, and the median OS was 9.3 months compared to 6.6 months [9]. The FRESCO-2 trial, which included a broader international patient population, confirmed these findings, further establishing fruquintinib as a valuable option for heavily pretreated mCRC patients [9]. These trials also tracked all side effects in both the fruquintinib and placebo groups [9].

Fruquintinib is generally well tolerated, with a well-documented safety profile from various clinical trials. Common side effects include hypertension, hand-foot syndrome, fatigue, and gastrointestinal disturbances. Specifically, hypertension occurs in approximately 21-30% of patients, and hand-foot syndrome is reported in about 20-30% of cases. Fatigue and gastrointestinal disturbances, such as diarrhea and nausea, are also frequently observed, with incidences ranging from 20-25% for fatigue and 15-20% for gastrointestinal issues [10]. More severe adverse events, although less common, include gastrointestinal perforation, which has been observed in about 1-2% of patients, and non-specific hemorrhage, occurring in approximately 2-3% of cases [10]. These severe events, while rare, are significant due to their potential for life-threatening complications. Notably, fruquintinib has been associated with serious hemorrhagic events, such as the cerebellar hemorrhage observed in this case.

While some targeted therapies are associated with an increased risk of secondary malignancies, fruquintinib has not shown a significant correlation with secondary cancer development in clinical trials [10]. Given these risks, it is crucial for clinicians to monitor patients closely, particularly those with predisposing factors for hemorrhage or other severe complications. The balance between the drug's benefits in controlling advanced cancer and its potential risks must be carefully considered in each patient’s treatment plan.

Beyond mCRC, fruquintinib is being actively investigated in other types of solid tumors, reflecting its potential broader applicability in oncology. Notably, the FALUCA trial is a phase III, randomized, double-blind, placebo-controlled study evaluating fruquintinib in Chinese patients with advanced nonsquamous non-small-cell lung cancer (NSCLC). Although it did not meet its primary endpoint of OS improvement, the study provided valuable insights into the drug's safety and efficacy in NSCLC, warranting further research [9]. Additionally, the FRUTIGA trial is a phase III study examining the combination of fruquintinib with paclitaxel in patients with advanced gastric cancer. This trial is expected to provide critical data on the drug’s effectiveness in another challenging cancer type [10]. For renal cell carcinoma, the FRUSICA-2 trial is a phase II study assessing the efficacy and safety of fruquintinib in combination with sintilimab, a programmed death-1 (PD-1) inhibitor, in patients with advanced renal cell carcinoma. This trial aims to explore the potential synergistic effects of combining VEGFR inhibition with immune checkpoint blockade, which could offer a new therapeutic avenue for this patient population [11].

These ongoing trials highlight fruquintinib’s potential broader applications beyond mCRC, offering hope for new treatment options in advanced cancers like NSCLC, gastric cancer, and renal cell carcinoma. The expanding scope of fruquintinib’s use underscores the importance of continued research to fully realize its therapeutic potential across multiple cancer types.

Fruquintinib offers significant benefits for metastatic colorectal adenocarcinoma but comes with risks, including rare yet serious complications like cerebellar hemorrhage. The proposed mechanism for such hemorrhagic complications involves VEGFR inhibition, which disrupts vascular integrity and angiogenesis, weakening blood vessel walls, particularly in the cerebellum, making them more susceptible to rupture. Additionally, VEGFR inhibition impairs the repair of damaged endothelial cells and compromises the blood-brain barrier, further increasing the risk of bleeding [11].

The cerebellum is particularly vulnerable to hemorrhage due to its dense and intricate vascular network, which includes a high concentration of small, delicate blood vessels. This region is also subject to significant blood flow, making it more susceptible to the effects of VEGFR inhibition, which can exacerbate the fragility of these vessels. The combination of these factors makes the cerebellum a critical area of concern when using therapies like fruquintinib that impact vascular stability [11].

Fruquintinib’s high selectivity for VEGFR1, VEGFR2, and VEGFR3 may lead to distinct effects on the vasculature in highly vascularized regions like the cerebellum, which is especially vulnerable due to its dense and delicate vascular network. The rarity of this side effect, despite the drug’s mechanism, may be influenced by individual patient factors such as genetic predispositions, co-existing conditions, or concurrent medications that exacerbate vascular fragility [11]. This case underscores the importance of thorough risk evaluation, especially for patients with cerebrovascular disease or conditions that increase bleeding risk. Clinicians must carefully balance these risks with the treatment's benefits, considering patient history and ensuring close monitoring to detect and address adverse effects early.

To minimize the risk of hemorrhage in patients receiving fruquintinib, a multifaceted approach is recommended. This includes a thorough baseline assessment to identify risk factors such as hypertension and previous cerebrovascular events, as well as careful screening for pre-existing conditions that might predispose patients to hemorrhage [2]. Controlling blood pressure aggressively is crucial, given the hypertensive and vascular weakening effects of VEGFR inhibitors, and regular monitoring of blood pressure and neurological status is essential to detect early signs of complications [6]. Medications that increase bleeding risk, such as anticoagulants, should be used with caution, and dose adjustments of fruquintinib may be necessary for high-risk patients [8]. Educating patients about the warning signs of hemorrhage and encouraging them to avoid activities that increase bleeding risk, such as trauma, injury, and strenuous physical activity, are also important preventive strategies. Additionally, regular follow-up and potential dose modifications can further reduce the risk of severe hemorrhagic events, making fruquintinib a safer option in a subset of patients for treating mCRC [11].

## Conclusions

This case underscores the critical importance of awareness and vigilance in managing the potentially severe complications associated with fruquintinib's selective targeting in left-sided descending metastatic colorectal adenocarcinoma. The development of cerebellar hemorrhage in this patient highlights the unique challenges of using fruquintinib in such cases, particularly when standard treatments have failed. While fruquintinib offers substantial clinical benefits, particularly in extending PFS for patients who have exhausted other treatment options, it also carries the risk of rare but life-threatening side effects, such as cerebellar hemorrhage, due to the combined effects of increased blood pressure and VEGFR inhibition. Although there are no specific criteria, clinicians must carefully assess the risk-benefit ratio for each patient, considering individual risk factors that may predispose them to hemorrhagic events. Ongoing research is essential to further elucidate the mechanisms underlying these complications and to develop strategies for their prevention and management, ultimately optimizing the safety and efficacy of fruquintinib in diverse patient populations.
